# Genitourinary cancer management during a severe pandemic: Utility of rapid communication tools and evidence‐based guidelines

**DOI:** 10.1002/bco2.18

**Published:** 2020-05-25

**Authors:** P. Shah, F. J. Kim, B. M. Mian

**Affiliations:** ^1^ Department of Urology Mayo Clinic Rochester MN USA; ^2^ Division of Urology University of Colorado Denver CO USA; ^3^ Division of Urology Albany Medical Center Albany NY USA

**Keywords:** bladder cancer, COVID‐19, pandemic, prostate cancer, Twitter

## Abstract

Objectives: To determine the usefulness of social media for rapid communication with experts to discuss strategies for prioritization and safety of deferred treatment for urologic malignancies during COVID‐19 pandemic, and to determine whether the discourse and recommendations made through discussions on social media (Twitter) were consistent with the current peer‐reviewed literature regarding the safety of delayed treatment. Methods: We reviewed and compiled the responses to our questions on Twitter regarding the management and safety of deferred treatment in the setting of COVID‐19 related constraints on non‐urgent care. We chronicled the guidance published on this subject by various health authorities and professional organizations. Further, we analyzed peerreviewed literature on the safety of deferred treatment (surgery or systemic therapy) to make made evidence‐based recommendations. Results: Due to the rapidly changing information about epidemiology and infectious characteristics of COVID‐19, the health authorities and professional societies guidance required frequent revisions which by design take days or weeks to produce. Several active discussions on Twitter provided real‐time updates on the changing landscape of the restrictions being placed on non‐urgent care. For separate discussion threads on prostate cancer and bladder cancer, dozens of specialists with expertise in treating urologic cancers could be engaged in providing their expert opinions as well as share evidence to support their recommendations. Our analysis of published studies addressing the safety and extent to which delayed cancer care does not compromise oncological outcome revealed that most prostate cancer care and certain aspects of the bladder and kidney cancer care can be safely deferred for 2‐6 months. Urothelial bladder cancer and advanced kidney cancer require a higher priority for timely surgical care. We did not find evidence to support the idea of using nonsurgical therapies, such as hormone therapy for prostate cancer or chemotherapy for bladder cancer for safer deferment of previously planned surgery. We noted that the comments and recommendations made by the participants in the Twitter discussions were generally consistent with our evidence‐based recommendations for safely postponing cancer care for certain types of urologic cancers. Conclusion: The use of social media platforms, such as Twitter, where the comments and recommendations are subject to review and critique by other specialists is not only feasible but quite useful in addressing the situations requiring urgent resolution, often supported by published evidence. In circumstances such as natural disasters, this may be a preferable approach than the traditional expert panels due to its ability to harness the collective intellect to available experts to provide responses and solutions in real‐time. These real‐time communications via Twitter provided sound guidance which was readily available to the public and participants, and was generally in concordance with the peerreviewed data on safety of deferred treatment.

## 1 INTRODUCTION

Since the initial reports in late December 2019 of respiratory illness caused by a novel coronavirus (COVID‐19) originating in Wuhan, China, the disease has made its way to over 200 countries.^1^ In a short span of less than 5 weeks from its initial report to the WHO, COVID‐19 infection was declared a public health emergency, and within 10 weeks, it was declared a global pandemic^2^, ^3^ on March 11, 2020. As of April 20, 2020, over 2 400 000 COVID‐19 cases had been reported globally, resulting in over 165 000 deaths.^4^ With such an exponential increase in COVID‐19 infections, many counties were caught unprepared for the massive demand on their healthcare system, including parts of China, Europe, and the USA. Our inability to perform wide scale testing to identify asymptomatic cases and perform appropriate contact tracing further accelerated the spread of COVID‐19.

The COVID‐19 pandemic exposed a universal lack of planning to deal with a highly contagious pathogen. It has created an unprecedented healthcare crisis and has demonstrated the potential to overwhelm large healthcare systems worldwide. The stark reality of shortages in viral test kits, personal protective equipment (PPE), intensive care beds, ventilators, and trained personnel was quite apparent. In the absence of effective treatments or vaccines, social distancing has been most effective tool to curb the rapid spread of COVID‐19. This concept of mitigative social distancing also applies to patients and healthcare workers. At the epicenters of the COVID‐19 pandemic (such as New York City, Wuhan, China or Italy), mandated cancelation of all nonurgent medical care is the only logical option. Even, in the regions that currently are not severely affected, a precautionary stance of reducing all nonessential medical care to maintain capacity in the system (supplies, personnel) to handle a projected surge in COVID‐19 infections is required. In certain situations, >80% of patients may fit into the category of nonurgent or elective care. While this approach is prudent, its open to interpretation as to what is considered “essential or necessary” medical care, leaving a large gray zone between emergency and elective care.

As the pandemic unfolded, it became abundantly clear that the public health authorities and professional organizations had not prepared any specific guidance for such an event. Due to its highly contagious nature, new evidence of infectious and epidemiologic characteristics of the virus was emerging so rapidly that many of the earlier COVID‐19 related recommendations regarding elective procedures became outdated within days. The Society of American Gastrointestinal and Endoscopic Surgeons (SAGES) and the European Association for Endoscopic Surgery (EAES) recommendations in response to COVID‐19 crisis were published on March 21, required updates within 8 days.^5^ An international, multi‐institutional editorial on triage of elective urological surgery was made public around March 18, but 1 week later, the authors commented that some of the recommendations may no longer apply.^6^, ^7^ The Association of Surgeons of Great Britain & Ireland published guidance on March 25 which essentially prohibited laparoscopic surgery due to concerns over escape of viral particle‐containing CO2 aerosol and potential exposure of the surgical staff. After questions were raised by fellow professionals about the rationale and data behind the statement, the inter‐collegiate guidance on surgical procedures required updates by April 5.^8^, ^9^

### 1.1 Rapid communication with experts via Twitter

**FIGURE 1:**
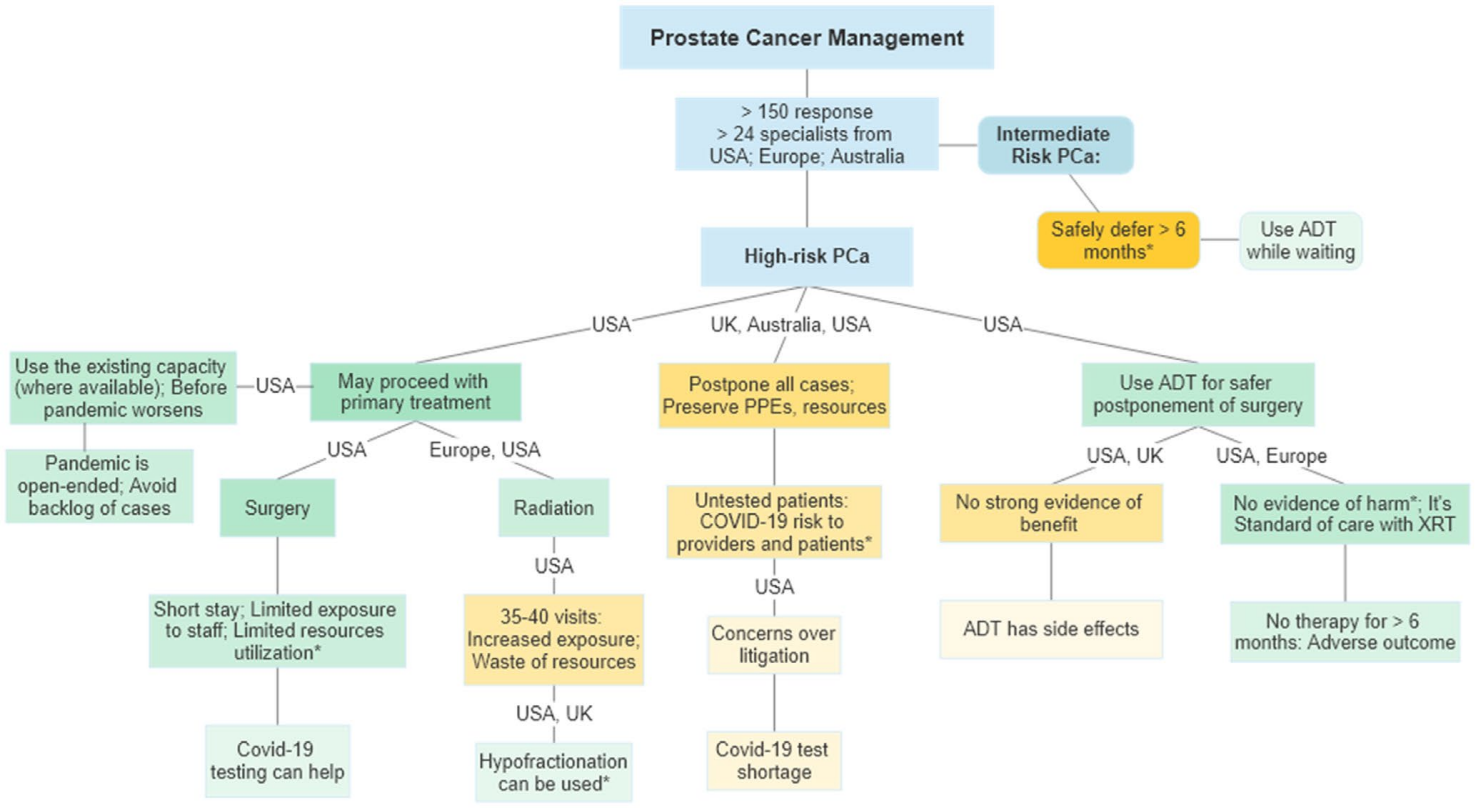
Frequency and strength of recommendations with the location of specialists responding to Twitter discussions about prostate cancer management in COVID‐19 constraints in regions other than the pandemic hotspots. *References provided by the respondents; Green: in support of treatment; Orange: in support of postponement. Darker shades: stronger or frequent comments

In the weeks after declaration of a global pandemic when governmental agencies and professional organizations were struggling to provide safe and practical recommendations, the responsibility for weighing the risk of COVID‐19 exposure to their patients (and staff) and the risks of deferred medical care was also taken up by the physicians. For rapid exchange of information and communication with fellow physicians and specialists with certain expertise, many were reliant on social media, especially Twitter.^6^ Herein, we will summarize the views, concerns and priorities expressed by many experienced specialists during three different discussions that took place on Twitter within 4 days. Discussions included practice patterns for prostate cancer (PCa) and bladder cancer management in the context of COVID‐19 related constraints on medical care. Some of the discussion and comments of the participants, with the strength or frequency of recommendations are presented graphically in Figure 1.

### 1.2 Prostate cancer

One of the earlier discussion was initiated by an urologist seeking opinions about prioritization of treatment of a 56 year old with multifocal high‐grade PCa, Grade group (GG) 4 and 3. The discussion was joined by a number of experts from various specialties with comments revolving around the timing and type of treatment, including the rationale for their particular approach. Within 1 day, there were over 60 responses from 15 specialists from the USA, Europe, and Australia. This discussion (and other discussions below) took place in mid‐March and should be viewed in the context of early stages of COVID‐19 in some countries relative to Italy and Spain (Table 1). It was nearly uniformly agreed that intermediate risk PCa treatment should be safe to defer for 6 months without any oncological harm.

**TABLE 1:** Estimated number of COVID‐19 infection on March 18, 2020

Country	Covid‐19 cases^a^
Italy	35,713
Spain	13,716
South Korea	8,413
United States	7,783
United Kingdom	676
Australia	568
Brazil	621

^a^ Source: www.statista.com.

For high‐risk disease, the rationale provided by some participants for proceeding with surgery was that, at the moment, the healthcare system is not overburdened, and a minimally invasive procedure will not excessively strain the resources. Some participants were adamantly opposed to any intervention in this scenario so that all PPE could be preserved for the potential surge in COVID‐19 cases. Others expressed concerns over the potential risk of COVID‐19 exposure during surgery to themselves and to the medical staff from an asymptomatic, untested patient. It was discussed that testing the patients for COVID‐19 virus before surgery could relieve some of these concerns, however, the tests are in short supply and not available for wider use. Some raised concerns that while it may be safe for a young patient with high‐grade cancer to wait 3 months, it's unclear how long the pandemic‐related restrictions are going to last. Their rationale to complete as much of the essential cancer treatment as possible before the anticipated surge in infections results in prolonged, open‐ended delays. The discussion was not limited to only the opinions of the experts. Their rationale was supported by providing peer‐reviewed literature and real‐time data such as availability of beds and PPE supplies from various medical centers.^10^, ^11^ The opinions, ranged from absolute no PCa surgery to cautiously proceeding with surgery, with daily assessment of the local situation in that hospital or region.

### 1.3 Change in practice pattern

**FIGURE 2:**
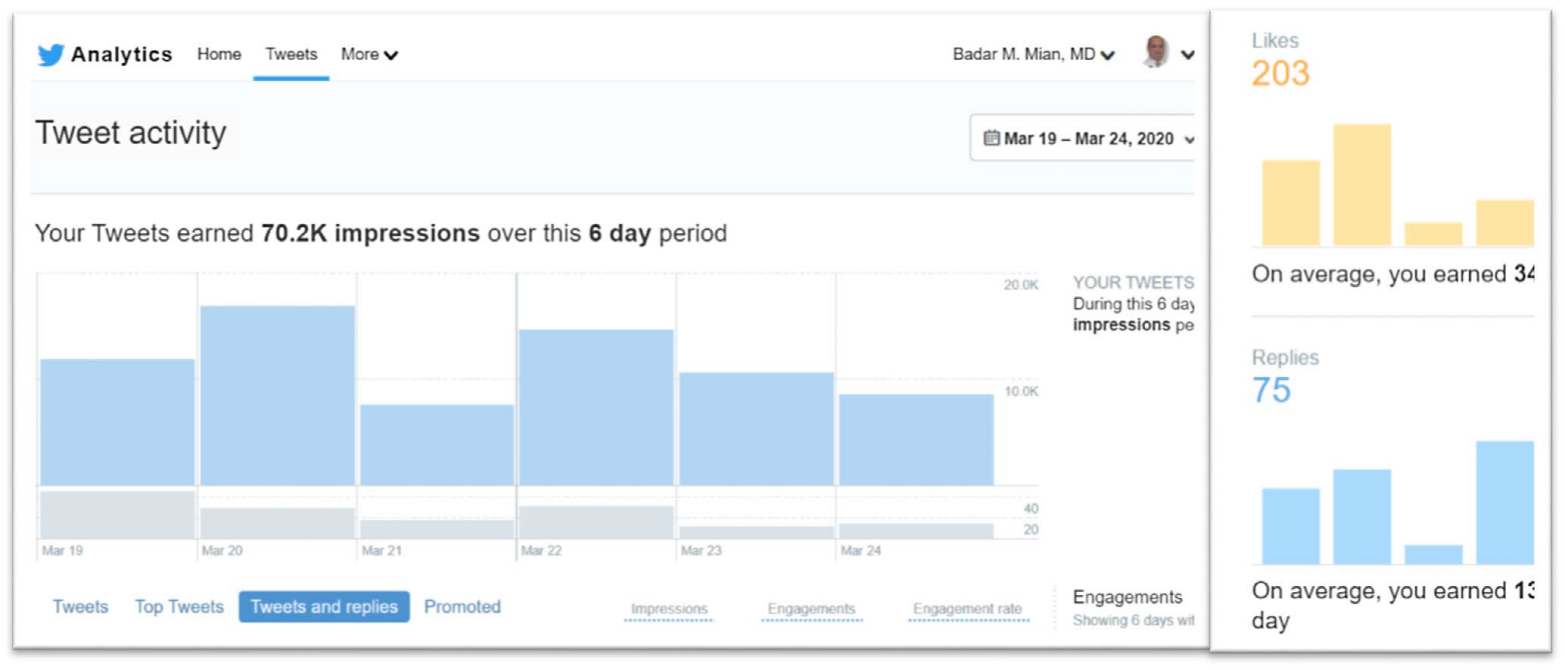
An example of the level of participation and engagement by multiple specialists in discussions regarding management of urologic cancer

Because of the variety of opinions noted during the above discussion, we asked urologic oncologists whether their own practice of PCa management had changed, or will change, due to COVID‐19. Specifically, would they consider delaying PCa surgery or consider radiation therapy (RT) instead of surgery or would they consider adding androgen deprivation therapy (ADT) when delaying surgery. In addition to the initially invited 12 urologic oncologists, the discussion was soon joined by additional specialists including radiation oncologists from US and Europe. The discussants generally agreed that surgery for patients with intermediate‐risk PCa can be safely delayed for 6 months. Others opined that since the potential duration of this pandemic is unknown and for some patients, it may already be 3 months since diagnosis, waiting for another 3‐6 months could lead to adverse outcomes. This concern was further amplified for patients with high‐risk PCa. The proponents of delayed surgery pointed to publications that delayed surgery was not associated with increased risk of biochemical recurrence.^12^ This was rebutted by another participant that only 5% of patients in that study had high‐risk PCa and only 7% were delayed for more than 6 months. The question about using neoadjuvant ADT to make it safer to delay surgery was met with spirited discussion. Some viewed using ADT for delayed surgery as merely providing peace of mind for the patient and the physician, but without any oncological benefit, citing a lack of strong evidence of benefits of neoadjuvant ADT. It was suggested that using ADT for delaying surgery for high‐risk PCa, especially if the anticipated delay is going to be more than 3 or 6 months, is a safe intermediary which could make it easier to accept the delays. Concerns were raised about ADT‐related adverse effects which can be quite pronounced in some patients. Other respondents viewed neoadjuvant ADT as comparable to the current standard of care of using ADT before RT. They referenced a randomized trial showing no significant adverse effects from using short‐term neoadjuvant ADT before surgery.^13^ Use of RT was discussed as an alternative to surgery to avoid the oncological risks of delayed treatment and the risk of COVID‐19 to the surgical team. At a time when resource conservation and COVID‐19 exposure mitigation are overriding concerns, it was debated whether the use of medical resources and risk of exposure was higher during roughly 40 visits to the facility for RT as compared to overnight hospitalization for minimally invasive surgery. While radiation oncology colleagues offered changing the practice to hypofractionated RT in 20 fractions, many discussants (mostly urologic oncologists) did not view that approach as having a lower risk of exposure or resource utilization. Within 12 hours, the discussion thread was engaged over a thousand times, yielding more than 100 suggestions and comments from over two dozen specialists (Figure 2). Many participants suggested that for high‐risk PCa, surgery or RT may proceed if local circumstances are permissive. But if anticipated delay is more than 6 months from diagnosis of high‐risk PCA, some suggested that the use of ADT may be discussed for a possible, but unproven oncological benefit due to its well‐understood safety profile.

On March 16, a pre‐print of guidance from a group of medical oncologists was presented on Twitter on the timing of chemotherapy for MIBC. They suggested that NAC could be offered to allow safer postponement of surgery. The claim of safety of NAC lead several respondents to question the wisdom of giving immunosuppressive treatment during a highly infectious pandemic, especially a treatment which is not curative. Concerns were raised about losing the curative window of time if the pandemic worsens while receiving NAC. Another poll conducted by a group of GU medical oncologists on March 18 about anticipated changes due to COVID‐19 in the management of cisplatin‐eligible stage T3 bladder cancer. Nearly 60% of 78 respondents would use the standard protocol of NAC and surgery while 37% preferred either surgery alone or with adjuvant chemotherapy. This suggested that roughly 1/3 of the respondents would potentially change their practice of NAC due to COVID‐19.

### 1.4 Bladder cancer

A detailed discussion on March 20 was held to strategize about the safety and effectiveness of various therapeutic sequences to treat muscle invasive (MIBC) and non‐muscle invasive (NMIBC) bladder cancer. Questions were asked about the best course of action for a patient with MIBC who finished neoadjuvant chemotherapy (NAC) 1 month ago and was scheduled for radical cystectomy in 2 weeks. Should that surgery be postponed due to potential shortage of supplies or should the surgery proceed as scheduled, before there is a COVID‐19 surge resulting in delay of 3‐6 months? How best to quantify or justify the potential risk of COVID‐19 exposure in the perioperative period or potentially worse oncological outcomes seen from the delay? Several of the uro‐oncologists recommended proceeding with the definitive excision and utilize early recovery pathways for short hospitalization since the treatment offers the best chance at cure and delayed surgery is associated with worse survival outcomes.

In a follow‐up discussion on March 22, participants from various sub‐specialties were queried about their preferred therapeutic sequence for stage T2 MIBC including surgery and/or chemotherapy and/or RT. The discussants included a fair mix of urologists, medical oncologist and radiation oncologists from North America and Europe. Well over 100 responses within 1 day from dozens of experts about the rationale for their approach, and 320 votes were cast about the preferred initial management. Interestingly, 44% favored NAC alone (and defer surgery), 41% would proceed with radical cystectomy alone, and 12% choosing primary RT with chemotherapy. The recommendation for postponing radical cystectomy in favor of NAC was to mitigate the risk of COVID‐19 exposure in the perioperative period. This idea was predicated on the significantly high utilization of medical resources including PPEs, hospitalization and readmissions due to complications. It was pointed out that the bladder cancer patients meet all the criteria for being at higher risk for COVID‐19 infection that is, older age, malignancy etc. And since the majority of adult chemotherapy for solid tumors offers only modest benefit, including MIBC with 5% overall survival improvement, the risks related to NAC may not be justified.^14^ Some participants provided pre‐print of guidance developed at their institutions to support their rationale for surgery or NAC taking into account the frequent visits to medial facility for infusion, lab work, possible imaging, blood work, PPE utilization, anesthesia services, and hospital beds. Radical cystectomy is a major surgery with complicated postoperative course and need for readmission to the hospital in roughly 30% of cases. A published reference was shared showing that cisplatin‐based chemotherapy is also associated with ER visits or hospitalization rate of 10%‐20%.^15^

A frequently discussed topic during all discussions was testing the patients for COVID‐19 infection as a measure to safely triage the patients, especially prior to surgery. However, due to the shortage of available testing kits, this option was deemed impractical as a routine measure at that time.

### 1.5 Communication with international urologists

Since mid‐March several informal conversations were conducted via social media platforms including Facetime and WhatsApp with international urologists to obtain updates and learn how COVID‐19 crisis had affected their lives, including scheduling of elective surgery. We also inquired whether there were deployments of urologists to different areas of the hospital that is, Emergency Department, Intensive Care Units, and other inpatient departments.

#### 1.5.1 South Korea

South Korea saw its first confirmed COVID‐19 case on January 20. The rate of infection gradually moved to 30 by February 17. By February 29, 2020, 40 days after its first confirmed case 909 new cases were diagnosed. It became the second most infected country after China by early March.^4^ South Korea undertook a massive public and private sector effort to fashion a national response to the pandemic.^16^ According to communications with colleagues from the Korean Urological Association (KUA), Korea's drive‐through testing proved to be an ingenious measure to protect healthcare workers from exposure while quarantining asymptomatic patients. Also, the use of masks with social distancing by the general population had become the norm in South Korea early in the pandemic.

Interestingly, there was minimal interruption of elective surgery and no redeployment of Urologists to different areas of the hospitals was necessary.

#### 1.5.2 Brazil

On February 25, 2020, after a 61‐year‐old man from São Paulo who had returned from Lombardy region of Italy tested positive for the virus,^17^ the rate of COVID‐19 infections has been increasing steadily but still lagging the numbers seen in Europe and USA (Table 1). However, like most other countries, Brazil's healthcare system is not immune to the potentially ravaging effects of a surge in COBVID‐19 cases. So, on March 27, when there were less than 4000 cases of COVID‐19 cases, most state governors (not a federal mandate) imposed quarantines to prevent the spread of the disease. By April 12, nearly 22 000 cases had been confirmed in the Brazil, causing 1223 deaths. All elective surgery were postponed, and telehealth^18^ was used as the preferred method for healthcare delivery whenever appropriate. Also, the annual meeting of the Brazilian Society of Urology was postponed from August to November of this year. A group of Brazilian specialists proposed some guidelines, that may be more applicable to low‐ or middle‐income countries,^19^ especially focusing on the goal of preserving health related resources (supplies, personnel).

#### 1.5.3 Italy

After China, Italy hit the hardest in the early days of the pandemic. Within 1 week of the declaration of a pandemic, it had reported over 32 000 cases and 250 deaths due to COVID‐19 by 18 March 2020. The Italian government enforced a national lockdown when the epidemic appeared limited to Italy's north. Report from a large hospital in Bergamo, a province not far from Milan, revealed an alarmingly rapid spread of the infections which overwhelmed the healthcare system's capacity to function.^20^ Their large urology department had completely shut down by March 19 to divert resources, including anesthesiologists, available beds and equipment, to the increasing number of patients with COVID‐19.

Urologists were deployed to fill the gaps in the different areas of the hospitals due to the limited capacity of the healthcare system to treat COVID‐19 patients. Nearly 30% of the urology staff were assigned to the newly diagnosed COVID‐19 patients. And by March 19, 7 out of the 13 urologists in the department (53%) required isolation at home after having symptoms and a positive swab for COVID‐19. In Rome, several tertiary care hospitals were designated to receive only COVID‐19 patients transferring all non‐COVID‐19 patients to other hospitals. All elective surgery were canceled in majority of hospitals. Throughout the country, doctors and nurses have struggled with shortages of protective equipment, which have sometimes cost them their lives and may have further fueled the spread COVID‐19 virus in hospitals.

#### 1.5.4 International urology conferences

On March 6, 2020 the Board and Executive of the European Association of Urology announced the postponement of the 35th Annual EAU Congress to July 17‐21, 2020 due to the public health safety measures in relation to COVID‐19 that extended worldwide. Moreover, on March 12, 2020 the Dutch government decreed the cancelation of all events in the Netherlands with 100 or more participants confirming the correct decision to postpone the EAU annual meeting.^21^ In addition, on April 1, 2020 the EAU announced that the 35th Annual EAU Congress July, 17‐21, 2020 would be conducted virtually.^21^ According to the EAU, the Virtual EAU20 will host a series of interactive virtual meetings presenting all the highlights of EAU20 around the time that the congress was scheduled to take place. In the meantime, the EAU announced that the EAU20 abstracts and surgical videos will become available online [https://uroweb.org/].

Shortly after, on March 13, 2020, the AUA canceled the 2020 AUA Annual Meeting in Washington, DC. coupled with United States’ emergency declaration. They too announced a virtual meeting called Virtual Science—A New, Multi‐media Reinvention of the AUA2020 Poster & Podium Sessions.^22^ These are likely to be followed by cancelation of other societies and regional conferences. This will undoubtedly have a negative impact on the functions of professional societies, the research activities, the education of trainees, and on practitioners in both the academic and community setting for many months or even years to come. To mitigate some of the gap in educational activities many collaborative efforts have been initiated to provide distance learning via webinars and using surgical simulators to keep the trainees engaged in the learning process. These efforts are great for didactic learning but the senior trainees in their final year will definitely be deprived of hands‐on training as they look forward to opening a new chapter their careers as independent practitioners.

## 2 EVIDENCE FOR SAFETY OF DELAYED SURGERY

Recent policies pertaining to elective surgery, generally set forth by administrative bodies, are meant to serve the communal interests of the population over the interests of an individual. Current guidance statements on elective surgery utilize equivocal terms such as “non‐essential” or “non‐critical,” language that invites interpretation by individuals to identify those procedures needing prioritization vs those that may be safely delayed.^23^, ^24^, ^25^ Yet, the physicians must also attend to the needs of their individual patients and help them to navigate current circumstances with the least impact on their health. The ominous perceptions surrounding a cancer diagnosis invariably bring into question how care can ever be rationed from a medical and ethical standpoint. Fortunately, within the realm of urologic oncology, significant prognostic heterogeneity exists which affords considerable flexibility with respect to surgical scheduling.

Herein, we review the existing evidence, based on peer‐reviewed literature, for risk‐stratification surrounding the safety of deferred surgical management of urologic malignancies most frequently encountered by practicing urologists (Table 2).

**TABLE 2:** Prioritization of urologic cancer treatment during pandemic‐related limited access

Condition	Safe to Defer Therapy (Time)	Additional Considerations (strength of recommendation)	Surgical Urgency^a^
*Prostate cancer*			
Low‐risk	Indefinite	Active surveillance may be changed to watchful waiting without biopsy	None
Intermediate‐risk	>6 months	No changes to the planned management	Low
High‐risk	Up to 6 months	May discuss alternative such as RT or ADT (week)	Intermediate
Very high‐risk	3‐6 months	May offer neoadjuvant therapy in select cases (moderate)	Intermediate
*Kidney cancer*			
Small renal mass (≤4 cm)	>6 months	Surveillance; establish growth kinetics, using existing protocols (strong) Repeat imaging in 6 months	Low
Large renal mass (> 4 cm; T3) Asymptomatic	3‐6 months	Surveillance; establish growth kinetics. Repeat imaging in 3 months. Prioritize treatment if concerning growth rate > 0.7 cm/yr (strong)	Intermediate
Large renal mass symptomatic (bleeding, pain)	<2‐4 weeks	Renal or tumor embolization may allow additional time (week)	High
IVC tumor thrombus	<1 month	Prioritize Surgery	High
Metastatic	<1 month	Initiate systemic therapy (strong) Defer cytoreductive nephrectomy. Risk‐stratification, Response to therapy	Low
*Bladder cancer*			
Newly diagnosed mass: TURBT		Deferred resection: Risk of hematuria, clot retention, ER visit or admission, increase resource utilization	
Papillary, asymptomatic	1‐2 months		Intermediate
Solid, asymptomatic	1 month		Intermediate
Symptomatic (hematuria, pain)	Days	No alternatives	High
NMIBC: Radical cystectomy	2‐3 months	Longer delays associated with worse pathology and survival. Alternative intravesical agents may be tried (week).	Low
BCG‐refractory			
T1, High‐grade			
Asymptomatic			
T1, High‐grade + CIS	1‐2 months	Longer delays associated with worse pathology and survival.	Intermediate
Symptomatic			
MIBC: Radical cystectomy	2 months	NAC, with deferred surgery may be offered (week). Primary RT may be used if surgery is not desired (Intermediate). The facility should be isolated to mitigate the risk of Covid‐19 due to frequent visits.	Intermediate
Stage cT2			
Cisplatin‐ineligible or			
Increased risk of COVID‐19 (age, frailty, immunity)			
Stage ≥ cT3	1‐2 months	NAC with deferred surgery, if resources available to mitigate Covid‐19 (strong).	High
Cisplatin‐ineligible		Otherwise, proceed with surgery.	
After NAC for any stage	1‐2 months	Further delay can compromise survival benefit.	High
*Upper tract UC*			
Low‐grade: Endoscopic	3 months	Initial endoscopic ablation should be thorough to reduce the need for multiple repeat procedure (strong)	Low
Symptomatic			Intermediate
High‐grade or large	<3 months	Initial treatment should be the most definitive (strong). Avoid repeat endoscopic procedures.	High
Nephroureterectomy			
Partial ureterectomy			
*Testicular cancer*			
Testicular mass	2 weeks	Delays in orchiectomy associated with risk for metastasis and reduced cancer survival (strong)	High
Stage I	>3 months	Surveillance should be the primary management (strong). RPLND and chemotherapy should be avoided	Low
± High risk features			
Stage ≥ II	<2 months	Defer primary RPLND. Encourage use of chemotherapy (strong).	Intermediate
Post‐chemotherapy Retroperitoneal Mass	<1 month	RPLND (strong). High‐risk for tumor progression and reduced cancer survival. <3 cm mass: observation, imaging is an option	High

Note

The colors represent overall severity of the condition and the need for treatment, Green: least concerning; Yellow: Intermediate; Orange: most concerning. The strength of additional recommendation is given in parenthesis (weak; intermediate; strong)

Abbreviations: ADT: Androgen deprivation therapy; BCG: Bacillus Calmette–Guérin; HG: high grade; MIBC: Muscle‐invasive bladder cancer; NAC: neoadjuvant chemotherapy; NMIBC: Non‐muscle invasive bladder cancer; RT: Radiation therapy; TURBT: Transurethral resection of bladder tumor; UC: Urothelial cancer.

^a^Surgical urgency defined as the need to perform surgery in <1 month: high; 2‐3 months: Intermediate; >3 months: low

### 2.1 Prostate cancer

#### 2.1.1 Low‐risk disease

Current guidelines advise that patients with low‐risk prostate cancer (PCa) should strongly be considered for AS protocol.^26^, ^27^ Using such a strategy can spare over 66% of these patients any unwarranted treatment. Supporting these guidelines are several large prospective studies, including that by the University of Toronto, which revealed 10‐year and 15‐year metastasis‐free survival rates of 96% and 95%, respectively, among the low‐risk cohort.^28^ A similar analysis of very low risk and low‐risk patients placed on AS by the group at Johns Hopkins revealed cancer‐specific and metastasis‐free survival rates exceeding 99% at 10‐ and 15‐year follow‐up.^29^ A recent observational study indicates that outcomes on AS are essentially equivalent between men ≤60 vs >60 years old at a median 6.2‐year follow‐up, including rates of metastasis‐free survival (99.7% vs 99%) and cancer‐specific specific survival (100% vs 99.7%).^30^

Under current circumstances, it is ever‐more critical that urologist embrace the data supporting surveillance for low‐risk PCa. Counseling should be structured to not only suggest that delayed intervention is safe, but rather no intervention is necessary at all for vast majority of patients. Non‐surgical therapies such as focal ablations or RT should be similarly discouraged to avoid resource utilization and medical interactions. Adjuvant tools such as multiparametric magnetic resonance imaging or genomic tests can be integrated in AS protocols, when available, to reduce the number of biopsies and to reassure both patient and physician regarding the absence of adverse pathology.^31^

#### 2.1.2 Intermediate‐risk disease

The presence of low‐volume Gleason pattern 4 disease is a more concerning finding, given the likelihood for genetic aberrations that permit metastatic progression.^32^, ^33^, ^34^, ^35^ Among patients with grade group 2 PCa (eg, Gleason score 3 + 4 = 7) placed on AS, risk for metastasis at 15 years was found to have increased nearly fourfold,^28^ suggesting that among men without competing health risks, primary intervention may be preferred. However, the AS protocol used in this study was not very stringent (eg, long biopsy intervals) and it did not differ among men with GG1 and GG2 cancer. It is very possible that a more stringent surveillance protocol for men with GG2 would have captured those cases before metastases occurred and still offer the benefits of AS to majority of patients. However, for younger patients with multifocal GG2 cancer, treatment is recommended, but delayed treatment by over 6 months does not appear to compromise oncological outcomes. Thus, with the pandemic‐related constraints, almost all of the patients with GG2 PCa can have the treatment postponed, either surgery or RT, for several months if necessary.

#### 2.1.3 High‐risk disease

A more relevant question under current circumstances is about the optimal timing of intervention for high‐risk PCa, and whether delays in therapy will impact oncologic outcomes. Several studies have evaluated the impact of delayed treatment for patients with high‐risk disease.^36^, ^37^ A recent retrospective evaluation of 2,303 mean with GG 3 or higher clinically localized PCa compared pathologic and clinical outcomes among men who underwent radical prostatectomy within 3 months of diagnosis to those whose surgery occurred between 3 and 6 months after diagnosis.^37^ No significant differences were noted in need for adjuvant therapy after RP, 5‐year biochemical recurrence‐free or metastasis free survival between the groups. These findings broadly align with results from the Scandinavian PCa Group Study Number 4, which demonstrated a relatively protracted time interval between diagnosis of clinically localized PCa (at least 50% of which were high‐risk) and the development of metastasis.^38^ Studies evaluating delayed treatment for only GG 4 or 5 PCa are small and infrequent. When considered in aggregate, the current evidence suggests that primary intervention for PCa can be safely deferred for up to 6 months even among the heterogenous higher‐risk group. With some exceptions, such as the highest risk patients (cT3, GG 4‐5), this group need not be assigned surgical urgency when developing a schema for the prioritization of care of urologic oncology patients.

#### 2.1.4 Neoadjuvant ADT

ADT prior to RT for high‐risk PCa is considered a standard of care and has long‐established clinical efficacy and safety. Patients and physicians may understandably express concerns regarding deferred surgery for high‐risk and/or locally advanced PCa. They may seek neoadjuvant systemic treatment while awaiting definitive therapy, such as ADT which has been evaluated previoulsy.^39^ In fact, a review of the National Cancer Database reveals that the contemporary use of neoadjuvant hormonal therapy prior to radical prostatectomy has increased slightly over recent years, particularly for high‐risk cancer.^40^ It is important to note that a therapeutic benefit for neoadjuvant androgen deprivation has only been definitively demonstrated in the setting of primary radiation.^41^ Role of ADT before radical prostatectomy for high‐risk disease remains undetermined. While several trials have demonstrated a pathologic benefit to neoadjuvant treatment among surgical patients, specifically reduced rates of positive surgical margin,^42^ consistent improvement in recurrence‐free or cancer‐specific survival remains to be shown.^13^, ^43^ In circumstances prolonged delay in surgery is anticipated and concerns about oncological control exist, a short course of neoadjuvant ADT may be offered as a temporizing measure after careful consideration. It is unlikely that ADT prior to surgery for high‐risk PCa will results in any more significant adverse effects than those noted during ADT and RT.

### 2.2 Kidney cancer

#### 2.2.1 Small renal mass

Several large studies have corroborated conspicuously low rates of systemic progression among patients with small renal mass (SRM; (≤4 cm) managed expectantly.^44^, ^45^ The Renal Cell Consortium of Canada trial published in 2011 reported metastases in only 1.1% of patients at a median 28‐month follow‐up, thus offering high‐level prospective evidence justifying an initial trial of surveillance for SRM.^46^ Theses findings are consistent with the results of a more recent prospective study which demonstrated a cancer‐specific survival of 100% with AS for SRM.^47^

Thus, in times of deferred elective surgery, physicians should integrate surveillance strategies for SRM into decision‐making algorithms.^48^ For patients in whom primary treatment for SRM is recommended or desired (younger, healthier) these data serve to provide reassurance to patients and physicians that deferred intervention for over 6 months is highly unlikely to compromise survival outcomes.

#### 2.2.2 Inferior vena cava tumor thrombus

On the opposite end of kidney cancer spectrum are renal cell carcinomas exhibiting tumor thrombus extension into the renal vein or inferior vena cava. These tumors should be regarded as high‐risk because without treatment, the median survival for these patients is only 5 months and 1‐year cancer‐specific mortality over 70%.^49^ Treatment delays pose potential for tumor thrombus propagation, which not only increases the complexity of the surgery, but also heightens the risk for venous thromboembolic events to as high as 6% prior to surgery.^50^ As durable survival has been demonstrated by many series evaluating oncologic outcomes following surgery, the care of patients with RCC with inferior vena cava tumor thrombus should be prioritized.^51^, ^52^, ^53^

#### 2.2.3 Large renal mass

Management of clinically localized renal masses that fall in between these two extremes has a more intermediate level of urgency. Such tumors, often referred to as large renal masses, are staged as cT1b when between 4 and 7 cm in size and cT2 when greater than 7 cm. As these lesions are conventionally managed with up‐front surgery,^48^ series detailing the natural history with deferred intervention are often limited to elderly individuals with competing health risks, thus obscuring an accurate assessment of progression potential. A study of 68 patients with cT1b renal masses (median size 4.9 cm) who were followed for at least 6 months after diagnosis demonstrated a median linear growth rate of 0.44 cm/year, which is greater than the typical growth rate of SRM. Moreover, among patients who underwent delayed intervention, particularly for higher linear growth rates (median 0.72 cm/year), no progression to metastasis was appreciated at a median 32‐month follow‐up.^54^ Indeed, linear growth rate may be the key to identifying more aggressive large renal masses that would require earlier intervention to forestall progression. A separate study detailing the natural history of untreated cT1b tumors found that lesions which progressed to metastasis had a linear growth of 0.9 cm/year compared to 0.67 cm/year for tumors that eventually remained stable.^55^ With pandemic limitation in access to elective surgical care, the data indicate that an initial short period of surveillance, with interval imaging at 3‐6 months, is safe^56^ and would permit an understanding of tumor growth kinetics so as to allow proper triaging of patients with respect to need for immediate vs deferred intervention.

#### 2.2.4 Metastatic (synchronous) renal cancer

Unique to renal cell carcinoma among all the urologic malignancies is the accepted use of nephrectomy in the setting of synchronous metastatic disease. Several studies suggest a survival benefit derived from cytoreductive nephrectomy among properly selected, “favorable” risk patients. Suggested criteria include relatively low metastatic burden, disease in favorable organs sites (eg, lungs, adrenal), and suitable performance status.^57^, ^58^ However, the doctrine surrounding up‐front cytoreduction has been recently challenged by recent randomized trials that failed to demonstrate survival benefit. The CARMENA trial compared primary cytoreductive nephrectomy (followed by sunitinib) to sunitinib treatment alone among intermediate and poor‐risk patients with metastatic RCC. The study demonstrated non‐inferiority of up‐front sunitinib vs surgery,^59^ suggesting that cytoreductive nephrectomy may have a limited role in the management of this patient cohort.

As the prognostic outlook of metastatic RCC has further improved with the introduction of novel immunotherapy agents,^60^ an emerging strategy has been to initiate systemic therapy in patients with metastatic RCC and assess tumor response. The demonstration of an objective disease response can be used as a litmus test to identify those cases where cytoreductive nephrectomy may offer benefit. Indeed, the SURTIME trial demonstrated that delaying cytoreductive nephrectomy to allow for early initiation of systemic therapy does not adversely impact disease progression or overall survival, and may in fact be a safer strategy.^61^ On intention‐to‐treat analysis, 28‐week progression‐free survival was 43% in the sunitinib followed by nephrectomy arm and 42% in the immediate nephrectomy arm, suggesting that delaying surgery for a few months while receiving systemic therapy is safe^62^. Thus, with the pandemic‐related constraints in access to surgical care and hospital resources, cytoreductive nephrectomy can be safely deferred in favor of earlier initiation of targeted therapies.

### 2.3 Bladder (urothelial) cancer

#### 2.3.1 Muscle‐invasive disease

Muscle‐invasive bladder cancer (MIBC) is an aggressive disease that carries high risk for metastatic progression, including a relatively high incidence of relapse even after radical cystectomy.^63^ Given the association with occult metastases at time of diagnosis, the accepted standard of care currently involves administration of platinum‐based NAC based on evidence of 5% overall survival benefit and 9% disease‐free survival benefit, especially if initiated within 2 months of diagnosis.^64^, ^65^

Nevertheless, many patients are not candidates for NAC, particularly those with compromised renal function or unfavorable comorbid status. These individuals should be prioritized for radical cystectomy. Several studies have demonstrated that surgical delay of 2 or 3 months can adversely impact survival outcomes, thus allowing a narrow window of flexibility. ^66^, ^67^ In a study of patients undergoing upfront radical cystectomy, surgical delay ranging between 60 to 90 days from time of bladder cancer diagnosis to surgery or from time of transurethral resection to surgery were associated with a 34% and 18% increase in risk of death, respectively.^66^ For patients completingNAC, surgery should similarly be performed in a timely manner as delays in radical cystectomy have also been shown to confer poorer prognosis in this cohort.^67^

Its critically important that while we consider the sequence of treatment, we must also balance the direct risk to the patients from treatment, either upfront cystectomy or NAC, the frequency of medical encounters, the risk of readmission or ER visits, and the risk of COVID‐19 infection in the perioperative period or during 3‐4 cycles of cisplatin‐based chemotherapy. In this regard, we must carefully consider the net survival benefit from NAC, which appears to be relatively modest, and cystectomy which is the primary curative treatment. Radical cystectomy requires hospitalization for an average of 6 days and utilizes additional resources such as anesthesia services and PPEs, with roughly 25%‐30% risk of readmission.^68^ Use of NAC requires multiple medical encounters, for infusions and laboratory testing, requiring multiple PPEs for each encounter. As noted above, cisplatin‐based chemotherapy regimens are associated with immunosuppression, neutropenia, chemo‐induced GI symptoms all of which result in ER visits and hospital admission in up to 20% of patients.

Further, the demographics of MIBC patients are of particular concern for COVID‐19 infection because, even at baseline, these include all of the high‐risk features including age, co‐morbidities, and malignancy. Thus, adding immunosuppressive therapies such as cisplatin‐based NAC will increase the risk of acquiring COVID‐19 infection. A computational oncology modeling study estimated that the now well‐established age‐related COVID‐19 case fatality rate (CFR) could increase by 3‐ to 12‐fold in patients receiving chemotherapy.^14^ The authors estimated that with a 5% benefit from chemotherapy, the age‐related baseline CFR for a 70‐year patient who acquired COVID‐19 infection would be higher than the 5% benefit from chemotherapy and could increase threefold by adding the effects of cancer and chemotherapy. Thus, using NAC to defer surgery may not the best option for most patients. Combination of RT and chemotherapy has been used quite successfully, especially in European centers, and are likely less immunosuppressive than cisplatin‐based NAC. While the protocols vary, however, these bladder‐preservation protocols are no less labor‐intensive as these require dozens of visits to the hospital and are challenged by the same factors mentioned above for NAC.

In treating MIBC, the guiding principle is that we should employ the most effective single therapy first which provides the best survival outcomes. Both NAC and adjuvant chemotherapy provide important but limited survival benefit, thus may be offered on case by case basis to those who are strong candidate to receive cisplatin. RT and chemotherapy can be offered as an alternative, if cystectomy is not desired or possible, especially if these can be delivered in facilities that are separate from the main hospitals to reduce personal contacts and COVID‐19 exposure. The desired sequence of treatment is subject to the COVID‐19 related constraints placed at hospitals in that region, at that moment in time.

#### 2.3.2 Non‐muscle invasive disease

Non‐muscle invasive bladder cancer (NMIBC) is a more heterogenous entity, sub‐stratified further into low‐ and high‐risk groups. The cumulative incidence of progression to high‐risk NMIBC was only 8%, whereas that to muscle‐invasive disease was 1.8% over a median 7‐year follow‐up period.^69^ As such, in the absence of clinically significant symptomatology (eg, refractory hematuria, pain), elective transurethral resection of bladder tumor (TURBT) for papillary or small tumors can be deferred for some time, if necessary. Patients with NMIBC whose management may be most impacted by current restrictions are those with high‐grade, T1 disease. It is advisable that these patients undergo repeat TURBT as the risk for upstaging to muscle invasion may be as high as 40%.^70^ With that knowledge, it is imperative to perform a thorough, deep initial resection, with the intention to obviate the need for a repeat TURBT. If repeat resection is necessary, it can be deferred for up to 2 months.^71^

Patients with NMIBC in need of immediate surgical priority are those with persistence or recurrence of high‐grade T1 disease after BCG therapy. The 5‐year cumulative risk for muscle‐invasion among those undergoing a repeat course of BCG can be as high as 70%, with nearly 33% already having occult muscle invasion at the time of initial BCG failure.^72^, ^73^, ^74^ With the prognosis of secondary MIBC shown to be significantly worse than for primary MIBC,^75^, ^76^ definitive surgery should be prioritized in this group as it is done for primary MIBC. BCG‐naïve individuals with high‐grade T1 disease and concomitant carcinoma in‐situ (CIS),^74^ lymphovascular invasion, variant histology, or prostatic urethral high‐grade disease^77^ have an adverse clinical trajectory similar to the BCG unresponsive cohort. While a preferred approach by many experts is an induction course of intravesical BCG followed by repeat resection to risk stratify these individuals, the worldwide shortage of BCG supply may eliminate this option. Thus, these patients may also be prioritized for radical cystectomy.

Patients with persistent or recurrent CIS can be treated with a repeat induction course of BCG.^71^, ^78^, ^79^ However, BCG‐unresponsive CIS has an increased risk for progression to muscle invasive disease.^80^ Although the timeline for progression has not been clearly defined, several studies do indicate that subsequent bladder preservation attempts with salvage intravesical chemotherapy (ie, Valrubicin, Gemcitabine) do not necessarily compromise oncologic outcomes when compared to immediate radical cystectomy following BCG failure.^81^ As such, there likely exists a broader window for this cohort within which radical cystectomy can be performed compared to those with BCG‐unresponsive HG T1 disease.

### 2.4 Upper tract urothelial cancer

For high‐grade Upper tract urothelial cancer (UTUC), nephroureterectomy (NU) is performed with curative intent in most patients, although a select group of patients (solitary kidney, small distal tumor) can be managed with partial ureterectomy to preserve the renal unit. Mostly due the low incidence of high‐grade UTUC, there is scant literature on the adverse effect of delayed NU, but it suggests that delayed NU is associated with worse outcomes. Waldert et al noted that a delay in performing NU of more than 3 months was associated with worse stage and lymph node metastases when compared to those NU cases completed in <3 months from diagnosis.^82^ Another study analyzed the effect on overall survival due to incremental delays in performing NU for up to 180 days when compared to surgery performed within 30 days. They noted that the overall survival was similar if surgery was performed within a month or if it was delayed for up to 4 months. But delay of >4 months resulted in significantly worse overall survival when compared to those who underwent surgery within 1 month.^83^

While delay in performing ureteroscopic ablation of UTUC appears not have any adverse impact on survival parameters, the key question is whether repeat endoscopic (≥2) procedures to defer nephroureterectomy is a safer approach. Further, evidence suggests, similar to urothelial bladder cancer management, the initial treatment of high‐grade UTUC should be that which provides the most definitive long‐term cure that is, nephroureterectomy, without the need for repeat interventions. Multiple endoscopic, non‐curative procedures, with numerous interactions with healthcare workers, and increased resource utilization may place the patient at increased risk of oncological failure and COVID‐19 exposure.

### 2.5 Testicular cancer

#### 2.5.1 Stage I

It is not uncommon for patients to defer evaluation of a scrotal mass due to embarrassment or lack of awareness. It is conventional practice to perform radical orchiectomy within 1‐2 weeks of presentation to a urologist. Ozturk et al found that the median delay from symptoms to primary physician evaluation was about 30 days which was associated with worse pathologic stage (*P* = 0.01).^84^ Further, longer time between specialist referral and orchiectomy was associated with worse pathologic stage (*P* = 0.04). While the effect of delayed evaluation on overall survival is unclear, worsening cancer stage increases the intensity of treatment and surveillance protocol for these young men. Thus, orchiectomy should not be delayed for more than 2‐4 weeks, especially since these patients are typically young, healthy, at lower risk for COVID‐19 infection and the procedure can be performed on outpatient basis, sometimes with regional block instead of general anesthesia.

Active surveillance for Stage I testicular cancer is the accepted standard of care, with no significant difference in outcomes when compared to adjutant RT or chemotherapy.^85^ In the current pandemic‐related constraints, adjuvant therapies for stage I testis cancer, such as RT or RPLND, should be avoided in favor of surveillance.

#### 2.5.2 Metastatic germ cell cancer

Systemic chemotherapy is a well‐established curative treatment for advanced germ cell testicular cancer. While some of the concerns mentioned above about delivering chemotherapy (frequent interactions, repeated exposure) are applicable to this population, other issues are less relevant. These patients are typically younger, with few comorbidities, at lower risk for immunosuppression, and the survival benefits of systemic therapy are far greater than those noted for urothelial cancers. Because systemic therapy can be curative for advanced testis cancer and delayed therapy results in poor survival, delivering chemotherapy should be considered a priority for these patients.

Post chemotherapy residual retroperitoneal mass may be managed based on size and histologic criteria. Small mass (<3 cm) after chemotherapy, especially seminoma, is safe to observe and a period of monitoring with serial imaging can risk‐stratify these patients. Larger residual retroperitoneal masses require retroperitoneal lymph node dissection (RPLND) to consolidate the curative intent. Due to the low incidence of this disease, the data on the safety of decayed RPLND is scant but delays in surgical care, particularly exceeding 3 months, have been shown to significantly compromise survival.^86^ Due to the favorable demographics of this cohort (young, healthy), absence of alternative therapies at this stage, and the curative nature of these interventions, RPLND for residual mass should be prioritized.

## 3 SUMMARY

COVID‐19 pandemic is the fastest moving, most devastating pandemic witnessed by modern society. It has overwhelmed large healthcare systems in many countries requiring hard decisions regarding prioritization of health care. In countries that are bracing for a surge of COVID‐19 cases or expecting a prolonged pandemic, limitations have been placed on performing much of the nonurgent or nonemergent medical care to preserve necessary supplies, staff and equipment.

With the rapidly changing ground reality of COVID‐19 and its effect of health care systems, medical organizations have been trying to provide updated guidance as soon as possible. But by design, they require additional time to compile and disseminate the information. In the immediacy of a pandemic, many professionals relied on interactions with fellow professionals using social media, especially Twitter, for rapid exchange of ideas and information to stay apprised of the situation in various parts of the world. We reviewed our discussions with dozens of experts in the field of GU oncology, including urologists, medical oncologists, and radiation oncologists from several countries. The participants offered concise recommendations and perspective based on their clinical practice and local impact of COVID‐19. The narrative and recommendations were well thought out, supported by references, and often preceded similar recommendations made by health authorities and medical associations. The recommendations made during discussion on Twitter do not rise to the level of scientific evidence as these are considered only the opinions of the experts. It is noteworthy, however, that some components of the guidelines and recommendations published by professional societies are also, at time, based on expert opinion.

Interestingly, while performing our review of evidence‐based safety of deferred treatment, we found that the discussion and responses during Twitter discussions were generally concordant with the evidence‐based recommendations presented above. Thus, during times of crisis when urgent exchange of information is desired, real‐time discussions on a public forum, such as Twitter, with experts in their fields appears to be quite feasible and useful. Because of the open format, the responses are subject to rebuttal and critique. Such a discourse can potentially yield a robust set of recommendations.

Within GU oncology, there exists a sufficient degree of heterogeneity in cancer biology among our most commonly encountered malignancies. This allows us to prioritize the surgical (and non‐surgical) care, utilizing an evidence‐based approach for safely deferring certain treatment. Further, it is likely that deferred medical care will identify certain postponed interventions (ie, surveillance, frequency, radiology, labs) which resulted in no harm. These low value interventions may no longer be considered essential, requiring us to adjust the clinical guidelines. Our recommendations are subject to change as the landscape of COVID‐19 pandemic changes in different regions. As more accurate testing, effective treatment and vaccines become available, cancer care will be ramped up on a regional basis, depending upon the COVID‐19 burden at that time.
